# A single-camera video-based assessment of locomotive syndrome using pose-silhouette fusion model

**DOI:** 10.1371/journal.pdig.0001530

**Published:** 2026-06-26

**Authors:** Yu Moriguchi, Junichi Kushioka, Noriko Takemura, Hiroki Hagizawa, Masafumi Kashii, Ichiro Nakahara, Taichi Kimura, Keisuke Akiyama, Taku Fujimoto, Hajime Nagahara, Keiko Yamada, Rodrigo Navarro-Ramirez, Takenori Oda, Hideki Mochizuki, Ken Nakata, Seiji Okada, Satoru Tada

**Affiliations:** 1 Department of Orthopaedic Surgery, National Hospital Organization, Osaka Minami Medical Center, Kawachinagano, Osaka, Japan; 2 Department of Orthopaedic Surgery, Osaka University Graduate School of Medicine, Suita, Osaka, Japan; 3 Department of Medical Innovation, Osaka University Graduate School of Medicine, Suita, Osaka, Japan; 4 ayumo, Inc., Osaka, Osaka, Japan; 5 Osaka University Institute for Sports and Global Health, Osaka University, Suita, Osaka, Japan; 6 Department of Clinical Research, National Hospital Organization Osaka Minami Medical Center, Kawachinagano, Osaka, Japan; 7 Spine and Scoliosis Center, Shonan Fujisawa Tokushukai Hospital, Fujisawa, Kanagawa, Japan; 8 Department of Artificial Intelligence, Graduate School of Computer Science and Systems Engineering, Kyushu Institute of Technology, Iizuka, Fukuoka, Japan; 9 Institute of Datability Science, Osaka University, Suita, Osaka, Japan; 10 Department of Geriatric and General Medicine, Osaka University Graduate School of Medicine, Suita, Osaka, Japan; 11 Department of Liberal Arts, Faculty of Healthcare and Welfare, Saitama Prefectural University, Saitama, Japan; 12 Department of Rehabilitation Medicine, The University of Tokyo, Tokyo, Japan; 13 Department of Neurosurgery, Cleveland Clinic Florida, Weston, Florida, United States of America; 14 Department of Neurology, Osaka University Graduate School of Medicine, Suita, Osaka, Japan; 15 Department of Neurology, National Hospital Organization Osaka Minami Medical Center, Kawachinagano, Osaka, Japan; Xinjiang Medical University Affiliated First Hospital, CHINA

## Abstract

Locomotive syndrome (LS), characterized by declining mobility due to musculoskeletal disorders, significantly affects older adults and is highly prevalent in Japan. Early detection and intervention are crucial to mitigate disease progression and preserve quality of life. Current diagnostic methods rely on subjective self-reports and labor-intensive clinical assessments, underscoring the need for automated, objective, and scalable screening tools. Building on our previous work, this study presents an updated deep learning-based computer vision model for LS screening using single-camera walking videos. We collected 511 walking videos from 178 participants clinically classified into four LS stages (non-LS, stage 1, stage 2, and stage 3). We used the MMPose framework to extract 2D body keypoints and generated silhouette-based Gait Energy Images (GEIs) from seven frames automatically sampled from fixed 40-frame walking windows to capture complementary gait-shape information. Model performance was evaluated using 10-fold cross-validation based on held-out evaluation-window predictions, while ensuring that windows from the same participant and the same walking video were not split across training, validation, and test sets. We also evaluated an independent external validation cohort of 33 cases (LS0–1, n = 15; LS2–3, n = 18). In internal validation, the pose-estimation model achieved sensitivity of 0.9440 and an AUC of 0.9668, whereas the silhouette model showed specificity of 0.9085 and an AUC of 0.9191. Fusion models outperformed the individual approaches, with the highest F1-score (0.908) at a 60:40 pose-to-silhouette ratio and the highest AUC (0.971) at a 70:30 ratio. In external validation, the score-fusion model achieved 90.9% accuracy, 88.9% sensitivity, 93.3% specificity, a macro-F1 score of 0.909, and an AUC of 0.967. In conclusion, integrating pose-estimation and silhouette-based features substantially improves the accuracy and clinical utility of automated LS screening. This low-burden approach provides a practical bridge between population-level screening and definitive clinical assessment.

## Author summary

Many older adults gradually lose mobility because of musculoskeletal problems, a condition known in Japan as locomotive syndrome. Detecting this decline early is difficult in busy clinical settings because standard assessments require multiple tests and trained staff. We developed a video-based approach that uses a single side-view walking video captured with an ordinary camera. From each walking sequence, we derived two complementary representations from seven frames automatically sampled from a fixed 40-frame walking window: body keypoints estimated from pose tracking and a silhouette-based gait energy image that summarizes movement in the same sampled frames. We trained separate models on these features and then combined their output scores using decision-level fusion. Across internal validation, the fused model improved overall discrimination compared with either component alone, and an independent external validation cohort also showed high accuracy and class-separation performance. These results suggest that simple walking videos can support scalable screening and triage, potentially enabling earlier referral and intervention. This work provides a practical pathway toward non-contact, low-burden mobility assessment that could be deployed in outpatient settings and community screening programs.

## Introduction

Locomotive syndrome (LS) is a condition characterized by a decline in mobility function due to musculoskeletal disorders, affecting a significant portion of the population, particularly the elderly [1] and the previous research found that the mean prevalence of LS was 69.8% among the Japanese population [2]. As shown in Table 1, LS is generally evaluated in three stages according to diagnostic criteria (Table 1). As LS progresses and physical ability declines noticeably, it is considered physical frailty, known as “LS Stage 3,” where decreased mobility function hinders social participation [3,4]. Notably, a systematic review estimated that the global prevalence of physical frailty is 12% among individuals aged over 50 years [5], making early detection and intervention of LS crucial for mitigating the progression of LS and improving quality of life [1].

**Table 1:** Subtests for the LS risk test.

LS stage	Non-LS	Stage 1	Stage 2	Stage 3
Stand-up test	Able to stand up from a 40-cm chair on one leg	Unable to stand up from a 40-cm chair on one leg	Unable to stand up from a 20-cm chair with both legs	Unable to stand up from a 30-cm chair with both legs
Two-step test	≥1.3	<1.3	<1.1	<0.9
GLFS-25	≤6	≥7	≥16	≥24

Management strategies for LS include pharmacological treatments and surgical interventions for related musculoskeletal disorders, as well as physical rehabilitation aimed at enhancing muscle and balance strength [1]. Additionally, addressing symptoms such as pain and numbness, along with correcting nutritional imbalances, is part of a comprehensive approach to LS treatment [1]. LS is notable for its potential reversibility with appropriate intervention, highlighting the importance of prompt and accurate diagnosis [6]. While diagnosing LS may seem straightforward when following standardized charts, it requires subjective patient self-reports and clinical evaluations by healthcare professionals [7]. This labor-intensive and time-consuming process creates a gap in routine clinical diagnosis, hindering its widespread implementation. Consequently, there is a growing demand in the medical field for the development of an automated, objective, and cost-effective tool that could enhance the efficiency of LS screening and diagnosis, thus reducing reliance on manual processes.

In our previous study, we introduced a deep learning (DL)-based computer vision model for LS screening using single-camera walking videos [8]. This model, employing OpenPose for pose estimation and MS-G3D for spatial-temporal graph analysis with 186 walking videos by LS patients by a single side camera, demonstrated promising results in identifying individuals with LS.

While our initial study provided proof of concept for automated LS screening, it also highlighted areas for improvement. The model’s performance, particularly in terms of specificity, could be enhanced by increasing the size and diversity of the training dataset. Additionally, exploring alternative pose estimation methods and other feature extraction approaches, such as silhouette detection, could potentially lead to more accurate and robust LS detection through single-camera walking video [9,10].

In this study, we present an updated deep learning (DL)- based computer vision model for LS screening that addresses the limitations of our previous work. We have significantly expanded the training dataset by including a larger number of walking videos (from 186 in our earlier study [8], to 511 in this study), thereby ensuring a more comprehensive representation of gait patterns associated with LS. Furthermore, we have included an analysis of the fusion model performance to determine how many pose features should be mixed into the model to get the best performance.

We implemented several key modifications to the model architecture to further improve the accuracy and robustness of LS detection. First, we replaced OpenPose [11] with MMPose [12], an OpenMMLab-based pose-estimation framework for extracting 2D body keypoints from RGB video frames. Second, we diversified the feature extraction process by incorporating not only pose information but also silhouette-based gait energy images (GEIs) [13,14] analyzed with a residual convolutional network [15]. This multimodal approach was designed to capture complementary kinematic and body-shape-related gait characteristics relevant to LS, potentially leading to more accurate and robust screening.

## Results

### Demographics

Table 2 presents the stage-stratified baseline characteristics of the model creation cohort. Age increased progressively with locomotive syndrome severity, from 42.8 ± 15.1 years in the non-LS group to 76.8 ± 8.5 years in the stage 3 group (Kruskal–Wallis test, p < 0.001). In contrast, the proportion of female participants did not significantly differ across LS stages (chi-square test, p = 0.087). Body weight was not recorded in the original development cohort dataset; therefore, BMI was not available for stage-stratified analysis.

**Table 2:** Stage-stratified baseline characteristics of the model creation cohort.

LS stage	n	Age, mean (SD), y	Female, n (%)	Height, mean (SD), cm
Non-LS	63	42.8 (15.1)	44 (69.8)	160.4 (8.4)
Stage 1	51	57.3 (16.8)	35 (68.6)	161.4 (7.6)
Stage 2	19	72.5 (11.1)	8 (42.1)	160.4 (9.4)
Stage 3	45	76.8 (8.5)	25 (55.6)	156.4 (8.9)

### Model creation and internal validation

**Fig 1:**
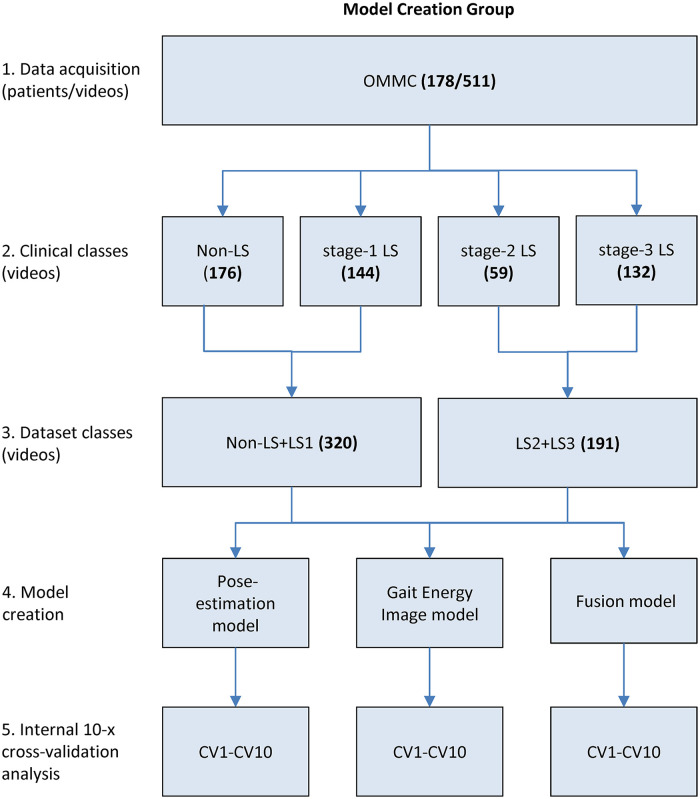
Data sets for model creation and internal validation. The model creation cohort comprised 178 participants (511 walking videos). Videos were categorized into four locomotive syndrome (LS) stages based on the standardized LS risk test (GLFS-25, two-step test, and stand-up test): non-LS (n = 176), LS stage 1 (n = 144), LS stage 2 (n = 59), and LS stage 3 (n = 132). For model development and evaluation, classes were binarized as non-LS plus LS stage 1 (n = 320) versus LS stage 2 plus LS stage 3 (n = 191). Multiple evaluation windows were sampled from the recorded 4- to 7-m walking segments for model training and evaluation. Pose-estimation, gait energy image (GEI), and fusion models were trained and evaluated using 10-fold cross-validation while keeping all windows from the same participant and the same walking video within the same split.

**Fig 2:**
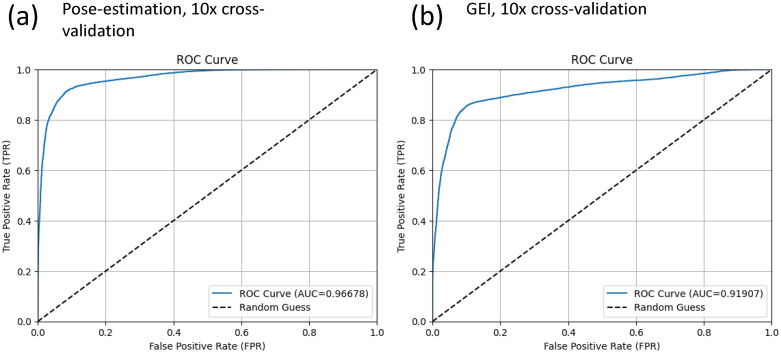
Receiver operating characteristic (ROC) curves for internal validation (10-fold cross-validation) of (a) the pose-estimation model and (b) the gait energy image (GEI) model for classifying non-LS plus LS stage 1 versus LS stage 2 plus LS stage 3 using held-out evaluation-window predictions. AUC values are shown in each panel.

The datasets used for model development and internal validation are described in Fig 1. Model performance was assessed using 10-fold cross-validation. The prediction records used for internal validation were automatically sampled seven-frame evaluation windows derived from the recorded 4–7 m walking segment. Multiple windows could be generated from one walking video for model training and evaluation; however, all windows from the same participant and the same walking video were assigned to the same split within each fold to avoid data leakage. In each fold, the data were divided into training, internal validation, and held-out test subsets using an 8:1:1 ratio. For the final 10-fold internal-validation results, held-out test predictions from all folds were pooled, and the reported performance metrics were calculated from these pooled evaluation-window-level predictions. The internal validation results are summarized in Table 3 and Fig 2.

**Table 3:** Internal validation by 10-fold cross-validation for the pose-estimation model and the gait energy image model using held-out seven-frame evaluation-window predictions.

	Sensitivity	Specificity	PPV	NPV	Accuracy	F1-score	AUC
pose-estimation model	0.9440	0.8475	0.8067	0.9574	0.8864	0.8700	0.9668
gait energy image model	0.8441	0.9085	0.8614	0.8964	0.8826	0.8527	0.9191

PPV; Positive Predictive Value, NPV; Negative Predictive Value, AUC; Area Under the Curve

### External validation

We additionally evaluated the models in an independent external validation cohort comprising 33 cases (LS0–1, n = 15; LS2–3, n = 18). In this cohort, the pose-estimation model achieved 81.81% accuracy, 83.33% sensitivity, 80.00% specificity, a macro-F1 score of 0.817, and an AUC of 0.922. The silhouette model achieved 84.84% accuracy, 83.33% sensitivity, 86.66% specificity, a macro-F1 score of 0.848, and an AUC of 0.930. The score-fusion model achieved the best overall external performance, with 90.90% accuracy, 88.88% sensitivity, 93.33% specificity, a macro-F1 score of 0.909, and an AUC of 0.967.

### Generation of a fusion model and evaluation of its performance by the ratio of the mixture of pose estimation

**Fig 3:**
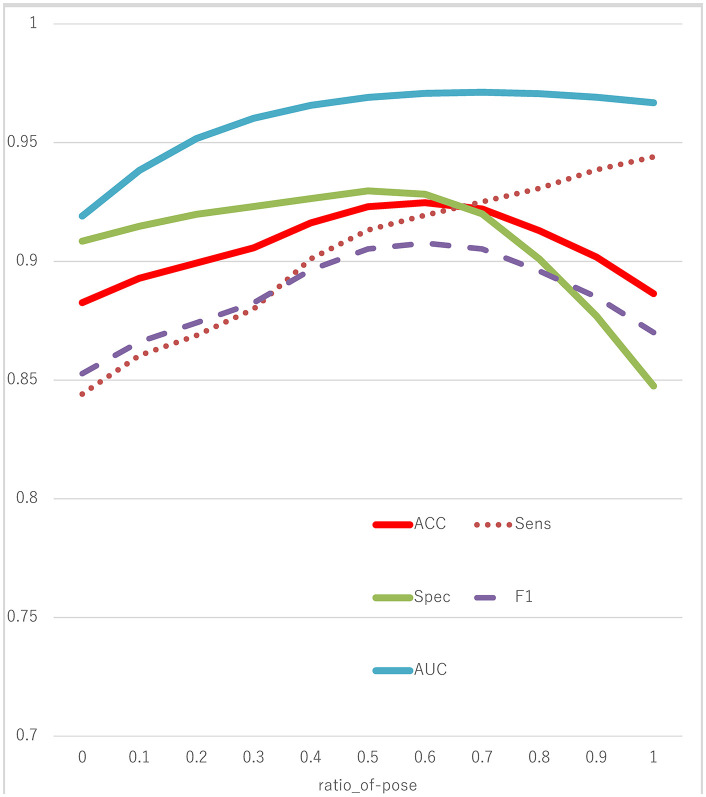
Performance of the fusion model as a function of the pose-estimation mixing ratio. The fusion score was computed as a weighted average of the pose-estimation model output score and the GEI model output score; the x-axis indicates the weight assigned to pose-estimation output (0 = GEI only; 1 = pose only). Accuracy, sensitivity, specificity, F1 score, and AUC are plotted for each mixing ratio using held-out evaluation-window predictions from 10-fold cross-validation.

**Fig 4:**
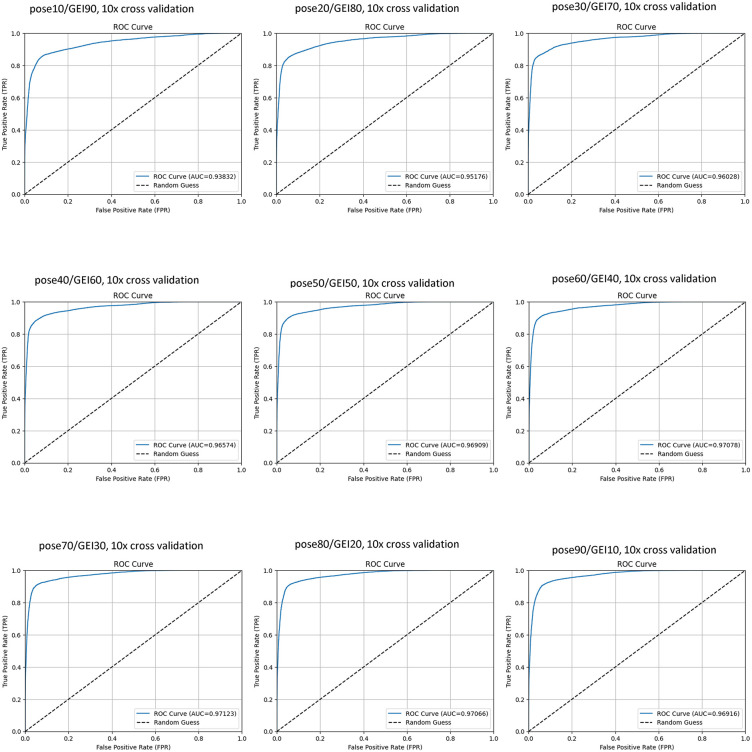
ROC curves of the fusion model for different mixing ratios of pose-estimation and GEI outputs (pose/GEI = 10/90 through 90/10). Each panel shows the ROC curve and the corresponding AUC from held-out evaluation-window predictions in 10-fold cross-validation.

We next evaluated the binary classification performance obtained by fusing the pose-estimation and silhouette models. As shown in Table 4 and Fig 3, diagnostic accuracy, sensitivity, specificity, F1-score, and AUC were assessed by varying the weight assigned to the pose-estimation output from 0.0 to 1.0 in increments of 0.1. Fusion models outperformed the individual approaches. The highest F1-score was achieved at a 60:40 pose-to-silhouette ratio (0.908, vs. 0.870 for the pose-only model and 0.853 for the silhouette-only model), whereas the highest AUC was achieved at a 70:30 ratio (0.971, vs. 0.967 and 0.919, respectively). Figure 4 illustrates the corresponding ROC curves for the different fusion ratios. Table 4 also reports false-positive and false-negative rates for each fusion setting, allowing the trade-off between missed LS2-3 cases and false-positive LS0-1 classifications to be evaluated according to the intended screening or triage use.

**Table 4:** Internal validation of fusion models by pose-to-silhouette ratio using held-out seven-frame evaluation-window predictions.

Pose:Silh	Acc	Sens	Spec	FPR	FNR	PPV	NPV	F1	AUC
1.0:0.0 (pose only)	0.886	0.944	0.848	0.153	0.056	0.807	0.957	0.870	0.967
0.9:0.1	0.902	0.939	0.877	0.123	0.061	0.837	0.955	0.885	0.969
0.8:0.2	0.913	0.931	0.901	0.099	0.069	0.864	0.951	0.896	0.971
0.7:0.3	0.922	0.925	0.920	0.080	0.075	0.886	0.948	0.905	0.971
0.6:0.4	0.925	0.919	0.928	0.072	0.081	0.896	0.945	0.908	0.971
0.5:0.5	0.923	0.913	0.930	0.070	0.087	0.897	0.941	0.905	0.969
0.4:0.6	0.916	0.901	0.926	0.074	0.099	0.892	0.933	0.896	0.966
0.3:0.7	0.906	0.880	0.923	0.077	0.120	0.885	0.919	0.883	0.960
0.2:0.8	0.899	0.869	0.920	0.080	0.131	0.879	0.912	0.874	0.952
0.1:0.9	0.893	0.860	0.915	0.085	0.140	0.872	0.907	0.866	0.938
0.0:1.0 (silh only)	0.883	0.844	0.909	0.092	0.156	0.861	0.896	0.853	0.919

Abbreviations: Acc, accuracy; Sens, sensitivity; Spec, specificity; FPR, false-positive rate; FNR, false-negative rate; PPV, positive predictive value; NPV, negative predictive value; AUC, area under the curve.

## Discussion

This study developed and validated a deep learning-based computer vision model for identifying clinically meaningful LS using single-camera walking videos. The updated framework combined pose estimation using MMPose [12], skeleton-based sequence modeling using MS-G3D [16], and silhouette-based gait representation using GEI [14] and ResNet18 [15]. Compared with our previous study [8], the current work used a substantially larger development dataset and incorporated external validation, leading to improved overall discrimination and more flexible tuning of sensitivity and specificity.

Compared with our previous study [8], the pose-based model benefited from the larger training dataset, and the complementary silhouette branch improved specificity. In the external validation cohort, the score-fusion model preserved high performance (accuracy 90.9%, AUC 0.967), supporting the robustness of combining these two representations in an independent binary cohort.

Silhouette and pose are widely used representations in gait analysis [17,18] and capture different aspects of gait. Pose representations emphasize kinematic relationships among joints and are relatively robust to appearance changes, whereas silhouette representations preserve holistic body shape and gait outline, which may carry clinically relevant information about body habitus and movement pattern. Consistent with prior studies combining pose and silhouette features [18], our fusion model achieved higher overall performance than either component alone.

This complementarity is clinically meaningful. Pose estimation reduces the human body to lines and keypoints, which can obscure shape-related information. In contrast, silhouette-based representations preserve global body contour and may reflect body habitus-related factors relevant to mobility impairment [19]. The observed improvement with fusion suggests that integrating kinematic and silhouette-derived features moves the system closer to the multifactorial clinical interpretation used in routine assessment.

We examined decision-level fusion across a range of pose-to-silhouette weights. Decision-level fusion combines the output scores of independently trained models, whereas feature-level fusion merges learned representations before the final classifier [20]. We selected decision-level fusion because it is simpler to implement, easier to tune, and computationally lighter than feature-level fusion, while still allowing complementary information from the two branches to be integrated; similar decision-level fusion approaches have been used in gait-based disease screening [21]. Although feature-level fusion may further improve performance, it increases network complexity and training burden.

The optimal fusion ratio depended on the intended clinical use. The highest F1-score was observed at a pose-estimation ratio of 0.6, whereas the AUC peaked at 0.7. These findings suggest that different operating points may be preferred depending on whether the emphasis is balanced classification performance or overall class discrimination. In practice, higher-sensitivity settings may be preferred for screening and triage, whereas higher-specificity settings may be more suitable when false positives should be minimized.

From a clinical implementation perspective, the proposed approach remains low burden, requiring only a side-view walking video captured in a 4 m × 10 m space with a single camera. This practical acquisition setting supports the potential use of the method in outpatient clinics and community screening environments.

The age distribution differed substantially across LS stages, with younger participants concentrated in the non-LS group and older participants concentrated in stages 2 and 3. This pattern reflects the epidemiology of LS, but it also raises the possibility that age-related gait characteristics contributed to model discrimination. Moreover, because the current dataset was derived from a Japanese population at a single center, differences in body morphology, gait characteristics, disease spectrum, and recording environments may limit generalizability to other populations.

This study has several limitations. First, although external validation was added, the external cohort was still limited in size and evaluated the same binary formulation (LS0–1 vs LS2–3); four-class classification performance was not assessed in the current study. Second, the reference labels were based on standardized clinical LS risk tests rather than objective biomechanical or longitudinal outcome measures. Third, body weight was not recorded in the original development cohort, and BMI could therefore not be analyzed in the stage-stratified baseline comparison. Fourth, the use of fixed 40-frame source windows with seven-frame sampling at six-frame intervals standardized the model input across participants, but it may not fully capture stride-to-stride variability or all gait-cycle characteristics in individuals with different walking speeds. Fifth, multiple evaluation windows were generated from each walking video for model development and internal validation; although the split strategy kept all windows from the same participant and the same walking video within the same split to prevent data leakage, the resulting evaluation-window-level predictions are not statistically independent at the walking-video level. Sixth, repeatability across repeated walking trials from the same participant was not formally quantified. Finally, inter-rater reliability of LS staging was not separately evaluated in this cohort.

## Methods

### Ethics statement

The study received approval from the Regional Committee for Medical and Health Research Ethics at National Hospital Organization Osaka Minami Medical Center (OMMC), ensuring compliance with ethical standards and patient safety. The Ethics Committee of OMMC granted specific approval for this prognostic study (Approval code: R5-42). Prior to inclusion, all OMMC participants provided written informed consent. All participants were adults aged 20 years or older; therefore, parental or guardian consent was not applicable.

The independent external-validation cohort included data from three sites. Data from Nose town residents were used under the approval of Osaka University Hospital (approval number: 19537-4), which covered walking-video acquisition, LS risk assessment, AI-based model evaluation, and secondary use for external validation.

The facility-based external-validation data from two long-term care facilities were collected with approval from the facility directors and with written informed consent from all participants or their authorized signatories. The facility directors determined that additional IRB review was not required because data collection did not use medical records or clinical care data and was limited to LS risk testing and walking-video recording for AI-based gait analysis.

The study protocol was designed to align with the principles outlined in the Declaration of Helsinki, guaranteeing respect for the rights and well-being of all participants.

#### Participants.

The study determined participant eligibility using specific inclusion and exclusion criteria. To be eligible for inclusion, participants were required to meet the following requirements: a minimum age of 20 years, voluntary participation, the ability to walk ten meters independently, consent to undergo the LS risk test, and undergo a medical examination by a certified orthopedic surgeon or neurologist. Individuals were excluded from this study if they were under the age of 20, unwilling to participate, or were unable to walk 10 meters independently.

For model development, a total of 178 patients who visited the Department of Orthopedics at OMMC between December 1, 2021, and March 30, 2022, were enrolled. Participants classified as non-LS were those who met the standardized LS risk-test criteria for non-LS. Internal validation was performed by 10-fold cross-validation using evaluation windows sampled from the walking videos, while ensuring that all windows derived from the same participant and the same walking video remained within a single split in each fold to prevent data leakage.

#### Data collection.

For the development of our model and internal validation, we gathered 511 walking videos from individuals enrolled in the study at the Department of Orthopedics at OMMC.

The video recording was conducted as previously described [8]. Briefly, participants were instructed to walk down a designated ten-meter path three times. Video recordings were obtained using the same camera system as in our previous study: a FLIR CHAMELEON3 camera (P/N: CM3-U3-13S2, Edmund Optics Inc., Barrington, NJ, USA) with a resolution of 1288 × 964 pixels, 30 frames per second, and 1.3 megapixels. An Edmund Optics UC fixed focal length lens (#33-300; 4-mm focal length, 12-megapixel C-mount lens) was used. During these walks, each participant was filmed from the right side, with the camera positioned four meters away from the walking route to ensure clear lateral movement capture. The raw footage was saved in MP4 format using Advanced Video Coding (AVC). To capture the stable walking phase, the 10-m walkway was physically marked at the 4-m and 7-m points before recording, and the camera field of view was adjusted so that only the 4- to 7-m segment of the walkway was visible in the recorded videos. Thus, participants walked naturally along the full 10-m walkway, but the recorded videos contained only the middle 4- to 7-m segment, excluding the acceleration phase at the beginning and the deceleration phase at the end. The 4- to 7-m walking segment was therefore defined by the physical walkway markers and camera framing at acquisition, not by post hoc localization using keypoints, timestamps, or algorithmic comparison with visual references. For technical reasons, some of the recorded videos could not be played back. In such cases, no more than two videos per participant were used as a training dataset.

#### LS risk test.

The LS risk test comprises three components: a patient-reported outcome measure called the GLFS-25, and two performance tests known as the two-step and stand-up tests (Table 1). These tests have been previously described in research papers [22] and were conducted as previously described [8]. The tests were administered according to the study protocol by trained research assistants and nursing staff. In brief, the GLFS-25 questionnaire consists of 25 questions, each rated on a Likert scale from 0 to 4, assessing difficulties related to mobility in daily life. Higher scores on this scale indicate a worsening health condition, and the total score, which ranges from 0 to 100, was used for analysis. Details of the GLFS-25 are shown in S1 Text. The two-step test involves patients starting from a standing position and taking two consecutive steps as far as possible. The distance covered by these two steps is standardized by dividing it by the patient’s height. This test is performed twice, and the best result is recorded. The stand-up test is conducted using stools of four different heights (10 cm, 20 cm, 30 cm, and 40 cm). Participants are required to stand up from these stools, either using one or both legs, and maintain their posture for 3 seconds after standing. A score between 0 and 8 is assigned based on successful performance, with a higher score indicating better physical condition.

The severity of LS is categorized using LS staging criteria as follows: normal, Stage 1 (the initial stage of decreased mobility defined by specific criteria for the two-step test, stand-up test, and GLFS-25 score), and Stage 2 (an advancing stage of decreased mobility defined by different criteria for the same tests). Additionally, a more severe stage known as Stage 3 (advanced decrease in mobility, limiting social engagement) has recently been defined, with specific criteria for the two-step test, stand-up test, and GLFS-25 score.

#### Deep learning-based locomotive syndrome prediction method.

Our deep learning-based method for predicting LS is described previously [8]. Briefly, it encompasses four main steps: (1) video recording, (2) pose estimation and silhouette estimation, (3) single and fusion model development, and (4) prediction of LS. The details of each step are described below.

1. Video recording and frame selection: We recorded subjects walking forward using a side-view camera setup that captured the predefined 4- to 7-m segment with clear visibility of the full body in motion. For model input generation, a fixed 40-frame walking window was used as the source window. From this window, seven frames were automatically sampled at six-frame intervals in 30-fps videos. This means that one frame was selected every six frames, leaving five intervening frames unselected; for example, if the starting frame was frame 1, frames 1, 7, 13, 19, 25, 31, and 37 were used. The same seven sampled frames were used to construct the pose-based and silhouette-based inputs. The six-frame interval was selected based on preliminary accuracy evaluation.
2. A) Pose estimation: The selected walking frames were processed using the MMPose framework [12] with the RTMW whole-body configuration rtmw-m_8xb1024-270e_cocktail14-256x192.py and checkpoint rtmw-dw-l-m_simcc-cocktail14_270e-256x192-20231122.pth. This public MMPose configuration uses a top-down pose-estimation pipeline with a CSPNeXt backbone, SimCC-based keypoint representation, input size 192 x 256, and 133 whole-body keypoints. The estimated keypoints were converted into the 25-body-keypoint representation used for the downstream MS-G3D classifier.
B) Silhouette estimation and generation of GEI: GEI [14] was used as a silhouette-based gait representation. A person-region segmentation method based on graph transfer learning [23] was applied to the seven sampled walking frames to generate silhouette images. Height normalization was then performed to obtain normalized silhouettes of 128 × 88 pixels. Finally, a single GEI of the same size was generated by averaging pixel values across the same seven normalized silhouette images sampled from the fixed 40-frame window. Although GEI is classically generated over one gait cycle, we used this fixed-window sampling approach because gait-cycle estimation can be unstable in patients with pathological gait and because the sampling interval had been selected based on preliminary accuracy evaluation.
3. A) Pose-estimation model development: The key point data derived from MMPose were converted into graph-structured format, representing joints as nodes and anatomical connections as edges. We then trained an MS-G3D-based classifier [16] to predict the binary LS label. For the skeleton-based model, batch size was 32. During model tuning, learning rates of 1e-3 and 1e-4 and weight decays of 1e-3 and 1e-4 were explored; the adopted parameters were learning rate 1e-3 and weight decay 1e-3.
B) Silhouette model development: The GEI inputs were processed using ResNet18 [15] to train a silhouette-based classifier. ResNet18 is a residual convolutional neural network that mitigates vanishing gradients by using skip connections. For the silhouette model, batch size was 256, learning rate was 1e-3, and weight decay was 1e-3.
C) Fusion strategy: To enhance performance, we implemented decision-level fusion. Two independently trained models—the pose-estimation model and the silhouette model—were combined by weighted averaging of their output scores. The fusion score was defined as the weighted mean of the two model outputs. To determine the most appropriate fusion setting, the pose-to-silhouette ratio was systematically varied from 0.0:1.0 to 1.0:0.0 in increments of 0.1.
4. Prediction of LS: The trained models output a prediction score for LS2–3 for each evaluation window. For binary classification, a threshold of 0.5 was applied; scores ≥ 0.5 were classified as LS2–3 and scores < 0.5 were classified as non-LS/LS1. Sensitivity, specificity, positive predictive value (PPV), negative predictive value (NPV), accuracy, false positive rate, false negative rate, F1-score, and AUC were calculated following the equations shown in S2 Text.

Processing time was measured for a representative approximately 6-second walking video on a workstation equipped with an Intel Core i9-14900KF CPU and an NVIDIA GeForce RTX 4090 GPU. In the skeleton branch, preprocessing with MMPose required 12.70 seconds and MS-G3D model inference required 0.28 seconds. In the silhouette branch, GEI generation required 45.35 seconds and ResNet18 model inference required 0.54 seconds. The final score-fusion step required 0.04 seconds. Because preprocessing time depends on the duration and content of the input video, these values should be interpreted as representative measurements for a 6-second sample video.

#### Statistical analysis.

Statistical analyses were conducted using Python 3.8, specifically leveraging the SciPy library for our calculations. Receiver operating characteristic (ROC) curves and the Area Under the Curve (AUC) were used to evaluate diagnostic performance during internal and external validation. For baseline comparisons among LS stages, Kruskal-Wallis tests were used for continuous variables and chi-square tests were used for categorical variables. For 10-fold internal validation, held-out test predictions from the evaluation windows across all folds were pooled, and sensitivity, specificity, positive predictive value, negative predictive value, accuracy, false positive rate, false negative rate, F1-score, and AUC were calculated from the pooled predictions.

## Supporting information

S1 Text GLFS-25. **The** 25-item Geriatric Locomotive Function Scale used for LS assessment.
(DOCX)

S2 Text Equations for diagnostic performance metrics. Definitions of sensitivity, specificity, positive predictive value, negative predictive value, accuracy, false-positive rate, false-negative rate, F1-score, and related evaluation metrics.
(DOCX)

S1 Data De-identified internal-validation model-output data. Minimal long-format prediction dataset containing random public row identifiers, binary true labels, fusion ratios, and model output scores for the internal-validation diagnostic performance analyses.
(CSV)

S2 Data De-identified external-validation model-output data. Minimal case-level dataset containing random public case identifiers, binary true labels, and pose, silhouette/GEI, and fusion model output scores/logits for the external-validation diagnostic performance analyses.
(CSV)

S3 Data Data dictionary. Descriptions and de-identification notes for the public tabular model-output data files.
(CSV)

S1 Code Reproduction script for internal-validation and external-validation diagnostic performance metrics. (PY)
