# Supplementary material for: A single-camera video-based assessment of locomotive syndrome using pose-silhouette fusion model
Source: PLOS Digit Health. 2026 Jun 26;5(6):e0001530. doi: 10.1371/journal.pdig.0001530 (PMC13308852; doi:10.1371/journal.pdig.0001530)
Supplement: S2 Text — Definitions of sensitivity, specificity, positive predictive value, negative predictive value, accuracy, false-positive rate, false-negative rate, F1-score, and related evaluation metrics. (DOCX) [file pdig.0001530.s002.docx]

S2 Text. Equations for diagnostic performance metrics

The following definitions were used to calculate diagnostic performance metrics. The positive class was LS stage 2 or 3, and the negative class was non-LS or LS stage 1.

| Metric | Definition or equation |
| --- | --- |
| True positives (TP) | Number of positive samples correctly classified as positive. |
| True negatives (TN) | Number of negative samples correctly classified as negative. |
| False positives (FP) | Number of negative samples incorrectly classified as positive. |
| False negatives (FN) | Number of positive samples incorrectly classified as negative. |
| Sensitivity | TP / (TP + FN) |
| Specificity | TN / (TN + FP) |
| Positive predictive value (PPV) | TP / (TP + FP) |
| Negative predictive value (NPV) | TN / (TN + FN) |
| Accuracy | (TP + TN) / (TP + TN + FP + FN) |
| False-positive rate (FPR) | FP / (FP + TN), equivalent to 1 - specificity |
| False-negative rate (FNR) | FN / (FN + TP), equivalent to 1 - sensitivity |
| F1-score | 2 x TP / (2 x TP + FP + FN) |
| Macro-F1 | Mean of the F1-score for the positive class and the F1-score for the negative class. |
| Area under the receiver operating characteristic curve (AUC) | Area under the ROC curve calculated from the model output scores/logits and the binary reference labels. |
